# SARS-CoV-2 and Dysphagia: A Retrospective Analysis of COVID-19 Patients with Swallowing Disorders

**DOI:** 10.1007/s00455-024-10715-0

**Published:** 2024-05-23

**Authors:** Christopher Molino, Laura Bergantini, Silvia Santucci, Marialuigia Tomai Pitinca, Miriana d’Alessandro, Paolo Cameli, Sabrina Taddei, Elena Bargagli

**Affiliations:** 1 Department of Medical Sciences, Surgery and Neurosciences, Respiratory Disease and Lung Transplant Unit, University Hospital of Siena (Azienda Ospedaliera Universitaria Senese, AOUS), Siena University, Viale Bracci, Siena, 53100 Italy; 2 U.O.P. della Riabilitazione, AOUS, Siena, Italy

**Keywords:** Dysphagia, Covid-19, Respiratory impariment, Rehabilitation, Speech-therapy

## Abstract

**Background**: COVID-19 can lead to impairment of neural networks involved in swallowing, since the act of swallowing is coordinated and performed by a diffuse brain network involving peripheral nerves and muscles. Dysphagia has been identified as a risk and predictive factor for the severest form of SARS-CoV-2 infection. **Objectives**: To investigate the association between swallowing disorders and COVID-19 in patients hospitalized for COVID-19. Methods: We collected demographic data, medical information specific to dysphagia and data on medical treatments of patients with COVID-19. **Results**: A total of 43 hospitalized COVID-19 patients were enrolled in the study. Twenty (46%) were evaluated positive for dysphagia and 23 (54%) were evaluated negative. Neurocognitive disorders and diabetes were mostly associated with patients who resulted positive for dysphagia. Respiratory impairment caused by COVID-19 seems to be a cause of dysphagia, since all patients who needed oxygen-therapy developed symptoms of dysphagia, unlike patients who did not. In the dysphagic group, alteration of the swallowing trigger resulted in the severest form of dysphagia. An association was found between the severest form of COVID-19 and dysphagia. This group consisted predominantly of males with longer hospitalization. **Conclusions**: Identification of COVID-19 patients at risk for dysphagia is crucial for better patient management.

**Supplementary Information:**

The online version contains supplementary material available at 10.1007/s00455-024-10715-0.

## Introduction

SARS-CoV-2 is a virus with single-stranded RNA. It belongs to the sub-family of Orthocoronavirinae, commonly known as coronavirus [1].

Several risk factors have been associated with COVID-19 severity. The best known are cardiovascular problems, diabetes, cancer, renal failure, smoking, obesity, history of transplant, immunosuppressive therapy and advanced age [2–9]. It has also been shown that neurological impairment occurs in almost 33% of patients infected with SARS-CoV-2, demonstrating that viruses of this family may cause persistent infection of cells of the central nervous system [10–15]. COVID-19 infection can also lead to impairment of neural networks involved in the swallowing process, since the act of swallowing is coordinated and performed by a diffuse brain network comprising cortical, subcortical and brainstem structures, and involving peripheral nerves and muscles [16–19]. The respiratory system is closely related to the swallowing system, and the consequences of the respiratory virus SARS-CoV-2 include alteration of breathing and swallowing coordination and consequently swallowing apnoea, both of which are essential for correct swallowing [19]. It is also presumed that the pathophysiology of oropharyngeal dysphagia in COVID-19 patients is linked to interaction of the virus with angiotensin-converting enzyme 2 (ACE2) and transmembrane serine protease 2 (TMPRSS2) [20], proteins present in anatomical regions specific to the swallowing function, such as the oral, pharyngeal and nasal mucosa. One of the main triggers of dysphagia, in that case, is impairment of the oropharyngeal sensory nerve, probably linked to glossopharyngeal and vagal sensory neuropathy [21–24].

Dysphagia has therefore been identified as a risk and predictive factor for pneumonia ab ingestis, dehydration, malnutrition, failed extubations, need for tracheostomy and prolonged mechanical ventilation in SARS-CoV-2 patients. Dysphagia associated with COVID-19 also correlates with longer hospitalization, higher mortality rate and worse outcomes [17–19, 22, 23, 25–31].

The aim of this study was to investigate the association between swallowing disorders and COVID-19 in patients hospitalized for COVID-19. The clinical characteristics of patients admitted to the COVID Unit who underwent swallowing assessment for suspected dysphagia were analysed for factors that could be involved in the onset of dysphagia and related to the severity of COVID-19. We also provide a description of swallowing in dysphagic our cohort of patients with COVID.19.

## Materials and Methods

### Study Design and Cohort

We enrolled 43 patients (24 males and 19 females, 73.7 +/- 14.1 years) in this monocentric retrospectively study. All had been admitted to covid units (medium or high intensive care or medical-surgical care) and had been referred for speech counselling in the period April 2020 to December 2022.

The following clinical and demographic parameters were considered: gender, age, length of stay in the covid unit, comorbidities, causes of dysphagia and previous intubation. During the period of speech-language assessment, intra-hospital pharmacological therapy, current oxygen therapy and related severity of respiratory impairment were also considered. Before doing food tests, all tracheostomized patients underwent the blue dye test to evaluate management of oral secretions. Subjects known to have dysphagia prior to admission were excluded.

Patients who tested positive for dysphagia were further evaluated, recording dysphagic symptoms after oral intake, modes of feeding after speech therapy assessment and related severity of dysphagia and compensatory/rehabilitative strategies. All subjects gave their written informed consent to participation in the study, which was approved by our Local Ethics Committee CEAVSE (PAN_HUB_2021 code number 17431_0_1). The study was performed in compliance with the Declaration of Helsinki.

### Swallowing Evaluation

Swallowing was assessed by Clinical Swallowing Examination (CSE), also known as Bedside Clinical Examination (BCE), which looks for the presence, nature and cause of dysphagia in the oral and pharyngeal phases of swallowing [32, 23], in order to confirm suspected dysphagia and make decisions regarding oral intake, consistency, additional compensations and the need for further examinations [34, 35]. The three-step evaluation consisted in recording clinical history, morphodynamic analysis of structures and organs involved in swallowing, and feeding tests with foods of different consistencies [36, 37]. Patients able to resume oral feeding were observed at mealtimes.

### Statistical Analysis

The results were expressed as means and standard deviations (M ± SD) or medians and quartiles (25th and 75th percentiles) for continuous variables, as appropriate. The Shapiro-Wilk test was used to test for normal distribution of the data. The Chi-squared test was used for categorical variables. One-way ANOVA non-parametric test (Kruskal–Wallis test) and the Dunn test or Friedman test were performed for multiple comparisons. The Wilcoxon test was used for paired variables. A *p*-value < 0.05 was considered statistically significant.

We also performed multinomial logistic regression to identify the variable that most influenced the severity of dysphagia. Statistical analysis and graphic representation of data were performed with GraphPad Prism 9.0 software (GraphPad Holdings, LLC, San Diego, CA, USA) and Jamovi Free software 2.6 version. A *p*-value < 0.05 was considered statistically significant.

## Results

### Cohort Stratification Based on Positivity or Negativity for Dysphagia

Twenty (46%) of the 43 Covid-19 patients enrolled in the study were referred for dysphagia screening; the other 23 (54%) were considered negative for dysphagia.

Our cohort of patients showed 7 mixed (oro-pharyngeal), 9 pharyngeal and 4 oral dysphagia.

Only two patients underwent to ORL evaluation due to the safety measures during COVID-19, but also due to the limited number of assessments for care management and for the limited possibility of requesting Ear-Nose-Throat (ENT) consultations.

For these 2 patients, who underwent to ENT evaluation, FEES in the first case gave more details about the dysphagic symptoms, in the second case it showed an edema of the arytenoids and false vocal folds.

No differences in gender distribution or days of hospitalization were detected. Patients referred for dysphagia screening were older that patients in the other group (not significant). Regarding comorbidities, neurocognitive disorders and diabetes were associated with patients referred for dysphagia screening. Regarding neurocognitive disorders, our cohort of patients was affected by cognitive impairment (25% of cases) and senil dementia (5% of cases). No differences in treatments, i.e. corticosteroids, sedatives and antiepileptics, were detected. A significant association was found between patients who did not require respiratory support during hospitalization and those considered negative for dysphagia (Table 1).

**Table 1:** Clinical treatment and type of therapy of the entire cohort of patients (*n* = 43) stratified by dysphagic/non-dysphagic

	Non dysphagic (*N* = 23)	Dysphagic (*N* = 20)	*p* value
**Gender**			0.61
M	12.0 (52.2%)	12.0 (60.0%)	
F	11.0 (47.8%)	8.0 (40.0%)	
**Age**			0.80
Mean (SD)	73.5 (15.8)	74.0 (12.3)	
Range	45.0–95.0	49.0–92.0	
**DAYS OF HOSPITALIZATION**			0.34
Mean (SD)	26.7 (21.3)	32.8 (21.5)	
Range	2.0–88.0	4.0–84.0	
**COMORBIDITIES**			
**NEUROLOGICAL**			0.95
NO	14.0 (60.9%)	12.0 (60.0%)	
YES	9.0 (39.1%)	8.0 (40.0%)	
**CARDIAC**			0.32
NO	14.0 (60.9%)	15.0 (75.0%)	
YES	9.0 (39.1%)	5.0 (25.0%)	
**RESPIRATORY**			0.43
YES	7.0 (30.4%)	4.0 (20.0%)	
NO	16.0 (69.6%)	16.0 (80.0%)	
**GASTROINTESTINAL**			0.17
NO	21.0 (91.3%)	20.0 (100.0%)	
YES	2.0 (8.7%)	0.0 (0.0%)	
**NEUROCOGNITIVE**			**0.02**
NO	23.0 (100.0%)	16.0 (80.0%)	
YES	0.0 (0.0%)	4.0 (20.0%)	
**DIABETES**			**0.07**
NO	21.0 (91.3%)	14.0 (70.0%)	
YES	2.0 (8.7%)	6.0 (30.0%)	
**PREVIOUS INTUBATION**			0.11
NO	22.0 (95.7%)	16.0 (80.0%)	
YES	1.0 (4.3%)	4.0 (20.0%)	
**TREATMENTS**			
**CORTICOSTEROIDS**			0.37
YES	17.0 (73.9%)	17.0 (85.0%)	
NO	6.0 (26.1%)	3.0 (15.0%)	
SEDATIVES			0.51
NO	16.0 (69.6%)	12.0 (60.0%)	
YES	7.0 (30.4%)	8.0 (40.0%)	
**ANTIEPILEPTICS**			0.88
NO	18.0 (78.3%)	16.0 (80.0%)	
YES	5.0 (21.7%)	4.0 (20.0%)	
**TYPE OF OXYGEN THERAPY**			
**HIGH FLOW**			0.10
NO	16.0 (69.6%)	9.0 (45.0%)	
YES	7.0 (30.4%)	11.0 (55.0%)	
**CPAP**			0.53
NO	20.0 (87.0%)	16.0 (80.0%)	
YES	3.0 (13.0%)	4.0 (20.0%)	
**NASAL CANNULA**			0.486
YES	8.0 (34.8%)	5.0 (25.0%)	
NO	15.0 (65.2%)	15.0 (75.0%)	
**NO OXYGEN SUPPLEMENT**			**0.027**
NO	18.0 (78.3%)	20.0 (100.0%)	
YES	5.0 (21.7%)	0.0 (0.0%)	

### Cohort Stratification Based on Severity of COVID-19

In order to detect a possible association of dysphagia with severity of COVID-19, patients were stratified as mild and moderate to severe (requiring tracheostomy or high oxygen flows) on the basis of need for respiratory support. Seventeen patients were considered mild and 26 moderate to severe. The onset of dysphagia was correlated with moderate to severe respiratory impairment. Interestingly, the mild group showed a prevalence of older women without dysphagia and with fewer days of hospitalization. Regarding treatments, greater use of sedatives and lower use of corticosteroids was associated with moderate-severe respiratory status (Table 2).

**Table 2:** Demographic data, comorbidities and treatments of patients stratified by severity of COVID-19

	MILD (*N* = 17)	MODERATE TO SEVERE (*N* = 26)	*p*-value
**GENDER**			< 0.01
M	5.0 (29.4%)	19.0 (73.1%)	
F	12.0 (70.6%)	7.0 (26.9%)	
**AGE**			**0.003**
Mean (SD)	81.4 (11.7)	68.7 (13.5)	
Range	60.0–95.0	45.0–92.0	
**DAYS OF HOSPITALIZATION**			**0.011**
Mean (SD)	19.5 (17.0)	36.1 (21.6)	
Range	2.0–60.0	10.0–88.0	
**DYSPHAGIA**			**0.015**
NO	13.0 (76.5%)	10.0 (38.5%)	
YES	4.0 (23.5%)	16.0 (61.5%)	
**COMORBIDITIES**			
**NEUROLOGICAL**			0.146
NO	8.0 (47.1%)	18.0 (69.2%)	
YES	9.0 (52.9%)	8.0 (30.8%)	
**CARDIAC**			0.101
NO	9.0 (52.9%)	20.0 (76.9%)	
YES	8.0 (47.1%)	6.0 (23.1%)	
**RESPIRATORY**			0.232
YES	6.0 (35.3%)	5.0 (19.2%)	
NO	11.0 (64.7%)	21.0 (80.8%)	
**GASTROINTESTINAL**			0.757
NO	16.0 (94.1%)	25.0 (96.2%)	
YES	1.0 (5.9%)	1.0 (3.8%)	
**NEUROCOGNITIVE**			0.128
NO	14.0 (82.4%)	25.0 (96.2%)	
YES	3.0 (17.6%)	1.0 (3.8%)	
**DIABETES**			0.899
NO	14.0 (82.4%)	21.0 (80.8%)	
YES	3.0 (17.6%)	5.0 (19.2%)	
**TRACHEOSTOMY**			**0.002**
NO	17.0 (100.0%)	15.0 (57.7%)	
YES	0.0 (0.0%)	11.0 (42.3%)	
**TREATMENTS**			
**CORTICOSTEROIDS**			**< 0.001**
YES	8.0 (47.1%)	26.0 (100.0%)	
NO	9.0 (52.9%)	0.0 (0.0%)	
**SEDATIVES**			**0.001**
NO	16.0 (94.1%)	12.0 (46.2%)	
YES	1.0 (5.9%)	14.0 (53.8%)	
**ANTIEPILEPTICS**			0.269
NO	12.0 (70.6%)	22.0 (84.6%)	
YES	5.0 (29.4%)	4.0 (15.4%)	

### Dysphagic Group Description

Severity of dysphagia was evaluated on the basis of feeding mode according to swallowing assessment, which could vary from non-oral feeding to different diets for dysphagic patients provided by the hospital dieticians. With this criterion it was possible to divide our cohort into mild (*n* = 4), moderate (*n* = 10) and severe (*n* = 6). No differences in gender or days of hospitalization were detected between the three groups, although patients in the severe group were older than those in the other two groups. Dysphagia symptoms did not appear to differ significantly between groups. Alteration of the swallowing trigger was borderline significantly associated with severe dysphagia.

Regarding postural compensation and proposed rehabilitation methods, a semi-seated position with the trunk at 90° and bent-head posture were associated with a higher frequency in the mild and moderate cohorts than in the severe group (suppl. Table 1).

When the cohort was divided on the basis of COVID-19 severity (mild and moderate to severe), dysphagic patients with moderate to severe COVID-19 were prevalently males and stayed longer in hospital (suppl. Table 2).

## Discussion

Patients with SARS-CoV-2 infection may range from asymptomatic to severe with acute respiratory impairment that may require invasive respiratory support. They are therefore assumed to be at risk for swallowing disorders [30]. Although dysphagia is considered a risk factor for severe COVID-19, little data is available on the association of dysphagia with clinical aspects of COVID-19 [38].

The present study investigated factors that might be implicated in the development of dysphagia in SARS-CoV-2 patients in order to assess the impact that COVID-19 may have on the onset of swallowing disorders. We also describe the clinical features related to swallowing disorders in patients with COVID-19 and concurrent dysphagia.

We found that the manifestation of swallowing disorders is associated with the simultaneous presence of neurocognitive disorders [39]. It is well known that neurocognitive disorders affect alertness during swallowing. Alertness is considered a fundamental prerequisite for correct oral intake of food. Impaired alertness may therefore be accompanied by the manifestation of dysphagia symptoms [40]. Alertness is considered a prerogative of swallowing disorders and not only of dysphagia associated with COVID-19.

Interestingly, postextubation dysphagia emerged as a common problem in recently extubated critically ill patients [41, 42]. Postintubation dysphagia during COVID-19 has also been suggested, although with limited support from the literature [43]. In our cohort of patients, previous intubation was a borderline significant factor for dysphagia. It was recently demonstrated that dysphagia is prevalent in COVID-19 patients after invasive mechanical ventilation and is associated with the number of days spent in hospital and the number of days in intensive care [21, 29, 30, 44–46].

Our results also showed that none of our COVID-19 patients who did not manifest respiratory impairment, and who consequently did not require oxygen therapy, were dysphagic. This suggests that the onset of dysphagia during SARS-CoV-2 infection could be related to underlying respiratory impairment caused by the infection itself, or conversely that the resolution of respiratory impairment involves simultaneous spontaneous resolution of the swallowing disorder. When we stratified our population as mild or moderate to severe on the basis of respiratory support, dysphagia showed a strong association with moderate to severe status, namely patients who required Ventimask, high-flow nasal cannulae or tracheostomy. This makes it plausible to assume that the severest patients are at highest risk of developing alterations in the swallowing process [47, 48].

We underline that, when patients were stratified by severity of respiratory impairment, including patients with high-flow cannulae and tracheostomy, tracheostomy itself can be regarded as a factor favouring dysphagia, although in our study it was mainly used as a criterion of severity of respiratory impairment due to COVID-19. In all tracheostomized patients, cannula dislodgement and secretion management tests were performed by the blue dye test, and only after these were passed were food tests performed.

The group of dysphagic patients with COVID-19 showed a prevalence of moderate and severe dysphagia.

Regarding the symptoms of COVID-19 dysphagia, we were unable to establish that the onset of one or more symptoms was specifically associated with the coronavirus infection except in a case of altered swallowing trigger and severe dysphagia. This association was borderline significant, possibly due to the poor coordination of breathing and swallowing typical of respiratory disorders, including SARS-CoV-2 [29].

Regarding postural compensation and rehabilitation methods, head and trunk flexed at 90° was proposed for all patients, irrespective of the severity of dysphagia. This was mainly due to the characteristics of COVID-19 hospitalized ward and the types of patient. These methods are the easiest to perform and are readily monitored by ward staff. Supraglottic swallowing was generally suggested for younger dysphagic patients; in our study, only one patient (younger than the average age of the study population) adopted this method.

Overall, our results propose a set of variables predictive of oropharyngeal dysphagia, and identify potential risk factors for hospitalized patients with COVID-19. Neurocognitive disorders and respiratory impairment caused by COVID-19 were associated with the onset of dysphagia. The dysphagia in these patients was moderate or severe. Severity of dysphagia was also associated with age.

Future research needs to determine dysphagia profiles in larger cohorts of patients who are evaluated for swallowing by fibro-endoscopy or video-fluoroscopy or by dysphagia severity scores. It is crucial for better management to identify medically fragile COVID-19 patients at risk of dysphagia.

## Electronic Supplementary Material

Below is the link to the electronic supplementary material.

Supplementary Material 1

## Data Availability

All data generated or analyzed during this study are included in this article. Further enquiries can be directed to the corresponding author.
