# Supplementary material for: SARS-CoV-2 and Dysphagia: A Retrospective Analysis of COVID-19 Patients with Swallowing Disorders
Source: Dysphagia. 2024 May 23;40(1):162–8. doi: 10.1007/s00455-024-10715-0 (PMC11762431; doi:10.1007/s00455-024-10715-0)
Supplement: Supplementary file 1 — Supplementary Material 1 [file 455_2024_10715_MOESM1_ESM.docx]

|  | **SEVERE (N=6)** | **MODERATE (N=10)** | **MILD (N=4)** | **p value** |
| --- | --- | --- | --- | --- |
| **AGE** |  |  |  | 0.138 |
| Mean (SD) | 81.3 (8.4) | 72.7 (13.2) | 66.0 (10.8) |  |
| Range | 69.0 - 92.0 | 49.0 - 90.0 | 58.0 - 82.0 |  |
| **DAYS OF HOSPITALIZATION** |  |  |  | 0.967 |
| Mean (SD) | 34.0 (31.1) | 33.0 (17.6) | 30.2 (19.2) |  |
| Range | 10.0 - 84.0 | 4.0 - 58.0 | 11.0 - 56.0 |  |
| **GENDER** |  |  |  |  |
| M | 5.0 (83.3%) | 5.0 (50.0%) | 2.0 (50.0%) |  |
| F | 1.0 (16.7%) | 5.0 (50.0%) | 2.0 (50.0%) |  |
|  |  |  |  |  |
| **REFLEX COUGH** |  |  |  | 0.430 |
| YES | 4.0 (66.7%) | 7.0 (70.0%) | 4.0 (100.0%) |  |
| NO | 2.0 (33.3%) | 3.0 (30.0%) | 0.0 (0.0%) |  |
| **SLOWED ORAL PHASE** |  |  |  | 0.207 |
| NO | 4.0 (66.7%) | 5.0 (50.0%) | 4.0 (100.0%) |  |
| YES | 2.0 (33.3%) | 5.0 (50.0%) | 0.0 (0.0%) |  |
| **IMPAIRED SWALLOWING** **TRIGGER** |  |  |  | 0.082 |
| YES | 3.0 (50.0%) | 1.0 (10.0%) | 0.0 (0.0%) |  |
| NO | 3.0 (50.0%) | 9.0 (90.0%) | 4.0 (100.0%) |  |
| **IMPAIRED SWALLOWING** |  |  |  | 0.435 |
| NO | 4.0 (66.7%) | 8.0 (80.0%) | 4.0 (100.0%) |  |
| YES | 2.0 (33.3%) | 2.0 (20.0%) | 0.0 (0.0%) |  |
| **WET VOICE** |  |  |  | 0.435 |
| YES | 3.0 (50.0%) | 2.0 (20.0%) | 1.0 (25.0%) |  |
| NO | 3.0 (50.0%) | 8.0 (80.0%) | 3.0 (75.0%) |  |
|  |  |  |  |  |
| **SEMI-SEATED POSITION** |  |  |  | < 0.001 |
| NO | 6.0 (100.0%) | 0.0 (0.0%) | 0.0 (0.0%) |  |
| YES | 0.0 (0.0%) | 10.0 (100.0%) | 4.0 (100.0%) |  |
| **FLEXED HEAD** |  |  |  | < 0.001 |
| NO | 6.0 (100.0%) | 0.0 (0.0%) | 0.0 (0.0%) |  |
| YES | 0.0 (0.0%) | 10.0 (100.0%) | 4.0 (100.0%) |  |
| **SUPRAGLOTTIC SWALLOWING** |  |  |  | 0.591 |
| NO | 6.0 (100.0%) | 9.0 (90.0%) | 4.0 (100.0%) |  |
| YES | 0.0 (0.0%) | 1.0 (10.0%) | 0.0 (0.0%) |  |

**Suppl. tab. 1** Postural compensation and proposed rehabilitation methods for dysphagic patients stratified by severity of dysphagia

|  | **mild (N=4)** | **mod-to-severe (N=16)** | **Total (N=20)** | **p value** |
| --- | --- | --- | --- | --- |
| **GENDER** |  |  |  | 0.01 |
| M | 0.0 (0.0%) | 12.0 (75.0%) | 12.0 (60.0%) |  |
| F | 4.0 (100.0%) | 4.0 (25.0%) | 8.0 (40.0%) |  |
| **AGE** |  |  |  | 0.134 |
| Mean (SD) | 82.2 (1.0) | 71.9 (13.0) | 74.0 (12.3) |  |
| Range | 81.0 - 83.0 | 49.0 - 92.0 | 49.0 - 92.0 |  |
| **DAYS OF HOSPITALIZATION** |  |  |  | 0.031 |
| Mean (SD) | 12.5 (6.5) | 37.8 (20.9) | 32.8 (21.5) |  |
| Range | 4.0 - 18.0 | 10.0 - 84.0 | 4.0 - 84.0 |  |
|  |  |  |  |  |
| **COUGH REFLEX** |  |  |  | 0.197 |
| YES | 2.0 (50.0%) | 13.0 (81.2%) | 15.0 (75.0%) |  |
| NO | 2.0 (50.0%) | 3.0 (18.8%) | 5.0 (25.0%) |  |
| **IMPAIRMENT ORAL PHASE** |  |  |  | 0.482 |
| NO | 2.0 (50.0%) | 11.0 (68.8%) | 13.0 (65.0%) |  |
| YES | 2.0 (50.0%) | 5.0 (31.2%) | 7.0 (35.0%) |  |
| **IMPAIRED DEGLUTITION TRIGGER** |  |  |  | 0.780 |
| YES | 1.0 (25.0%) | 3.0 (18.8%) | 4.0 (20.0%) |  |
| NO | 3.0 (75.0%) | 13.0 (81.2%) | 16.0 (80.0%) |  |
| **IMPAIRED DEGLUTITION ACT** |  |  |  | 0.264 |
| NO | 4.0 (100.0%) | 12.0 (75.0%) | 16.0 (80.0%) |  |
| YES | 0.0 (0.0%) | 4.0 (25.0%) | 4.0 (20.0%) |  |
| **WET VOICE** |  |  |  | 0.143 |
| YES | 0.0 (0.0%) | 6.0 (37.5%) | 6.0 (30.0%) |  |
| NO | 4.0 (100.0%) | 10.0 (62.5%) | 14.0 (70.0%) |  |

**Suppl. tab. 2** Postural compensation and proposed rehabilitation methods for dysphagic patients stratified by severity of COVID-19
